# The KEAP1–NRF2 pathway regulates TFEB/TFE3-dependent lysosomal biogenesis

**DOI:** 10.1073/pnas.2217425120

**Published:** 2023-05-22

**Authors:** Athena Jessica S. Ong, Cerys E. Bladen, Tara A. Tigani, Anthony P. Karamalakis, Kimberley J. Evason, Kristin K. Brown, Andrew G. Cox

**Affiliations:** ^a^Peter MacCallum Cancer Centre, Melbourne, VIC 3000, Australia; ^b^Sir Peter MacCallum Department of Oncology, The University of Melbourne, Melbourne, VIC 3010, Australia; ^c^Division of Anatomic Pathology, Department of Pathology, University of Utah, Salt Lake City, UT 84112; ^d^Huntsman Cancer Institute, University of Utah, Salt Lake City, UT 84112, USA; ^e^Department of Biochemistry and Pharmacology, The University of Melbourne, Melbourne, VIC 3010, Australia

**Keywords:** KEAP1, NRF2, lysosome, zebrafish, TFEB/TFE3

## Abstract

The maintenance of redox and metabolic homeostasis is integral to embryonic development. Nuclear factor erythroid 2-related factor 2 (NRF2) is a stress-induced transcription factor that plays a central role in the regulation of redox balance and cellular metabolism. Under homeostatic conditions, NRF2 is repressed by Kelch-like ECH-associated protein 1 (KEAP1). Here, we demonstrate that Keap1 deficiency induces Nrf2 activation and postdevelopmental lethality. Loss of viability is preceded by severe liver abnormalities characterized by an accumulation of lysosomes. Mechanistically, we demonstrate that loss of Keap1 promotes aberrant activation of transcription factor EB (TFEB)/transcription factor binding to IGHM Enhancer 3 (TFE3)-dependent lysosomal biogenesis. Importantly, we find that NRF2-dependent regulation of lysosomal biogenesis is cell autonomous and evolutionarily conserved. These studies identify a role for the KEAP1–NRF2 pathway in the regulation of lysosomal biogenesis and suggest that maintenance of lysosomal homeostasis is required during embryonic development.

## Significance

The KEAP1–NRF2 pathway plays a central role in the regulation of redox balance and cellular metabolism. Although NRF2 has been extensively investigated in various disease states, including cancer, there is a paucity of knowledge regarding the role of NRF2 during embryonic development. Here, we demonstrate that NRF2 activation induces lethality that is preceded by liver abnormalities and accumulation of lysosomes. Moreover, we find that NRF2 activates the master regulators of lysosomal biogenesis, TFEB/TFE3. These studies highlight a critical role for the maintenance of lysosomal homeostasis during embryonic development and, more broadly, suggest that aberrant lysosomal biogenesis may be a hallmark of NRF2-driven pathologies.

Maintenance of redox and metabolic homeostasis during growth and differentiation is critical for embryonic development. Studies performed in the 1930s, by Joseph Needham, were among the first to identify that cellular metabolism and redox state are dynamically regulated during embryonic growth (1–3). There is now a vast literature describing the deleterious effects of redox/metabolic perturbation during development (4). This is exemplified by inborn errors of metabolism, a devasting collection of congenital syndromes, which share in common the disruption of redox and/or metabolic homeostasis (5). Importantly, redox/metabolic perturbation during development can also be caused by environmental factors. For example, alcohol is among the most common environmental stressors and alcohol consumption underlies the development of fetal alcohol spectrum disorder (6). Despite the undeniable link between disruption of redox/metabolic homeostasis and developmental defects, the molecular mechanisms impacting development downstream of redox/metabolic perturbation are poorly understood.

NRF2 is a transcription factor that orchestrates the cellular response to redox imbalance (7). Under basal conditions, NRF2 is repressed by the redox-sensitive protein KEAP1 (8), which binds to the Neh2 domain of NRF2 to facilitate constitutive proteasomal degradation (9). Upon exposure to oxidative or electrophilic stress, the cysteine residues of KEAP1 are modified (10), which enables NRF2 to translocate to the nucleus and form a heterodimer with the small MAF proteins. NRF2 heterodimers bind specifically to antioxidant response elements (AREs) in the genome to induce target gene expression (11). Genome-wide ChIP-seq studies have identified hundreds of NRF2 target genes involved in a range of redox/metabolic processes, including glutathione (GSH) biosynthesis, nicotinamide adenine dinucleotide phosphate (NADPH) biosynthesis, heme metabolism, and autophagy (12–14).

NRF2 is dispensable for survival during embryogenesis (15, 16). In contrast, constitutive activation of NRF2 leads to postnatal lethality in mice (17). More specifically, the postnatal lethality observed in Keap1-deficient mice is preceded by hyperkeratosis of the esophagus and stomach resulting in malnutrition (17). More recently, clinical studies have identified patients with inborn activating mutations in NRF2 (*NFE2L2),* and these patients manifest with a multisystem disorder that involves a failure to thrive, immunodeficiency, and neurological symptoms (18).

In this study, we generated Keap1-deficient zebrafish to further investigate the role of NRF2 during development. Keap1-deficient larvae exhibit widespread activation of Nrf2 and concomitant abnormalities in the liver, which precede postembryonic lethality. At the molecular level, loss of Keap1 induces aberrant lysosomal biogenesis via activation of the microphthalmia transcription factor E (MiT/TFE) family of transcription factors, which includes TFEB and TFE3. Importantly, these features were also observed in mammalian cells, which illustrates the cell autonomous and evolutionarily conserved nature of this NRF2-dependent TFEB/TFE3 pathway.

## Results

### Loss of Keap1 Activates Nrf2 and Drives Postembryonic Lethality.

**Fig. 1.**
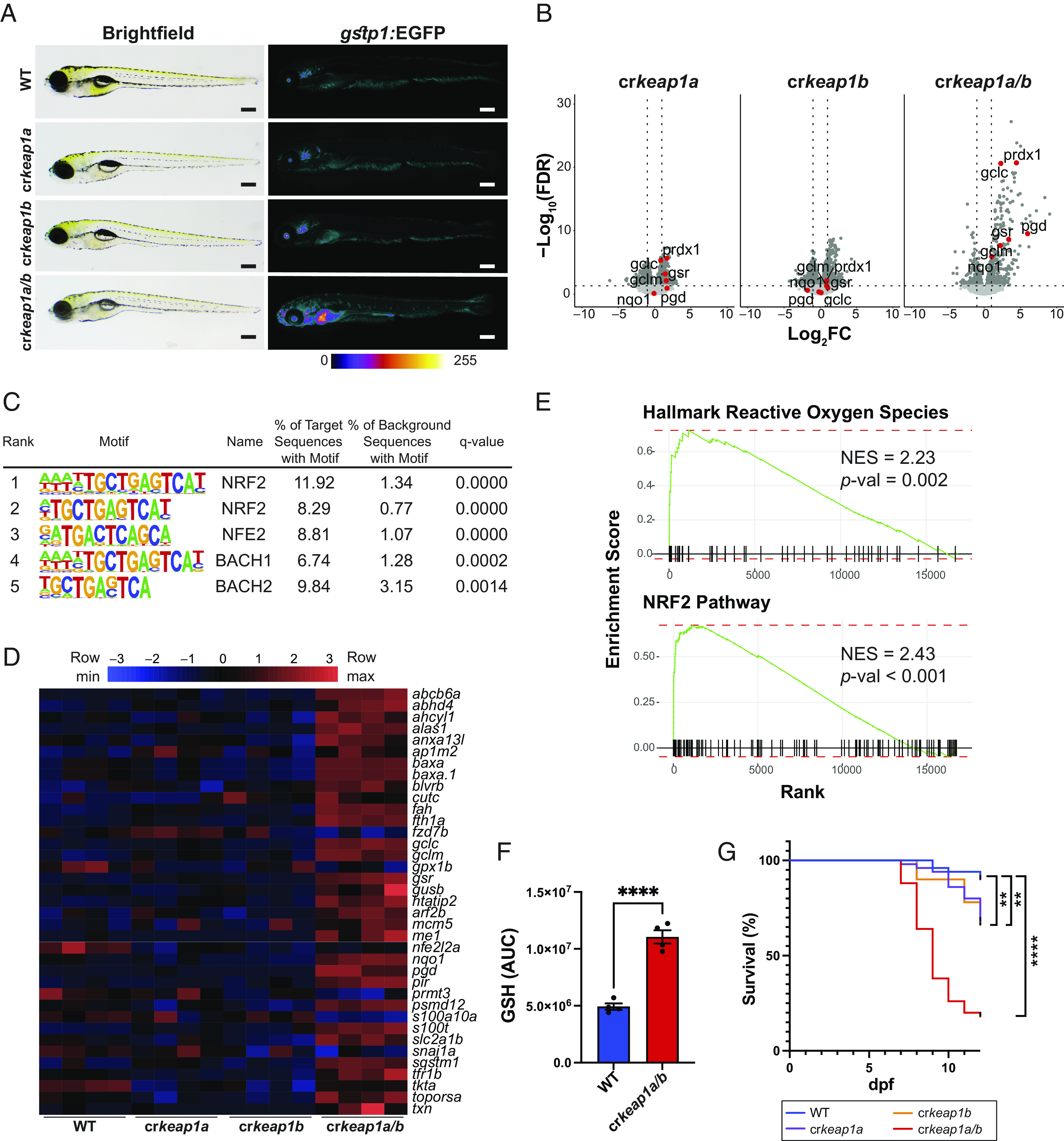
Loss of Keap1 activates Nrf2 and drives postembryonic lethality. (*A*) Representative whole-mount brightfield and fluorescent images of WT, cr*keap1a, *cr*keap1b*, and cr*keap1a/b* zebrafish on a *gstp1*:EGFP background at 7 days post fertilization (dpf). Fluorescent images are pseudocolored using the Fire Look-Up Table (LUT). Scale bars represent 350 µm. (*B*) Volcano plots of differentially expressed genes (DEGs) identified by comparing WT and cr*keap1a*, cr*keap1b*, or cr*keap1a/b* zebrafish at 7 dpf by RNA-Seq analysis, n = 4 pools of 10 larvae. Significant DEGs are highlighted in dark gray. Select canonical Nrf2 target genes are highlighted in red. (*C*) Top five enriched transcription factor-binding sites, as determined by Hypergeometric Optimization of Motif EnRichment (HOMER) motif analysis, among the genes up-regulated in cr*keap1a/b* zebrafish at 7 dpf. (*D*) Heatmap of Nrf2 target gene expression among DEGs identified in Fig. 1*B*. (*E*) Gene set enrichment analysis (GSEA) plots derived from RNA-Seq analysis of cr*keap1a/b* versus their WT counterparts at 7 dpf demonstrating Nrf2 pathway activation. (*F*) Glutathione (GSH) abundance in WT and cr*keap1a/b* zebrafish at 7 dpf as determined by LC-MS/MS. Data are shown as mean area under the curve (AUC) ± SEM, n = 4 pools of 10 larvae. (*G*) Kaplan–Meier survival plot of WT, cr*keap1a, *cr*keap1b*, and cr*keap1a/b* zebrafish, n = 50. For all experiments **P < 0.01, ****P < 0.0001.

In order to examine the role of Keap1 during embryonic development, a CRISPR/Cas9 gene editing approach was employed (*SI Appendix*, Fig. S1 *A–C*) (19). Due to genome duplication in the teleost lineage, zebrafish harbor two Keap1 paralogs (*keap1a* and *keap1b*) that act in a redundant manner with respect to regulation of Nrf2 (20). Crispants with mosaic knockout (KO) of *keap1a* (cr*keap1a*), *keap1b* (cr*keap1b*), or *keap1a/b* (cr*keap1a/b*) were initially generated in a Nrf2 reporter zebrafish line [Tg(*gstp1*:EGFP)] in which EGFP expression is driven by the ARE-containing *gstp1* promoter. No significant changes in Nrf2 reporter activity were observed following loss of *keap1a* or *keap1b* (Fig. 1*A*). In contrast, cr*keap1a/b* larvae exhibited increased Nrf2 activation (Fig. 1*A*). Nrf2 activation was not homogenous across tissues but was observed in neuromasts, olfactory cavities, otoliths, distal gut, and the liver. The Nrf2 activation phenotype was transmitted to the progeny of an in-crossed cr*keap1a/b* line (*SI Appendix*, Fig. S1*D*). Consistent with the lack of Nrf2 reporter activity, RNA-Seq analysis identified a limited number of differentially expressed genes (DEGs) in the single KOs (Fig. 1*B*). In contrast, widespread changes in the transcriptional landscape were observed in cr*keap1a/b* larvae (Fig. 1*B*). Hypergeometric Optimization of Motif EnRichment (HOMER) analysis of known motifs in the DEGs revealed enrichment of genes containing an ARE (Fig. 1*C*). Moreover, NRF2 target genes were overrepresented among the DEGs (Fig. 1 *B* and *D*). GSEA confirmed enrichment of signatures associated with NRF2 activation (Fig. 1*E*). Consistent with the recognized role of NRF2 in regulating GSH biosynthesis, metabolomics analysis identified a significant increase in the abundance of reduced GSH in cr*keap1a/b* larvae (Fig. 1*F* and *SI Appendix*, Fig. S1*E*). The molecular features associated with Keap1 deficiency preceded a marked loss in survival during postembryonic development (Fig. 1*G*), which is consistent with the postembryonic lethality observed in Keap1-deficient mice (17). These data demonstrate that compound loss of *keap1a* and *keap1b* in zebrafish provides a powerful model to investigate the role of the KEAP1–NRF2 pathway during development.

### Keap1-Deficient Larvae Exhibit Defects in Postembryonic Liver Development.

**Fig. 2.**
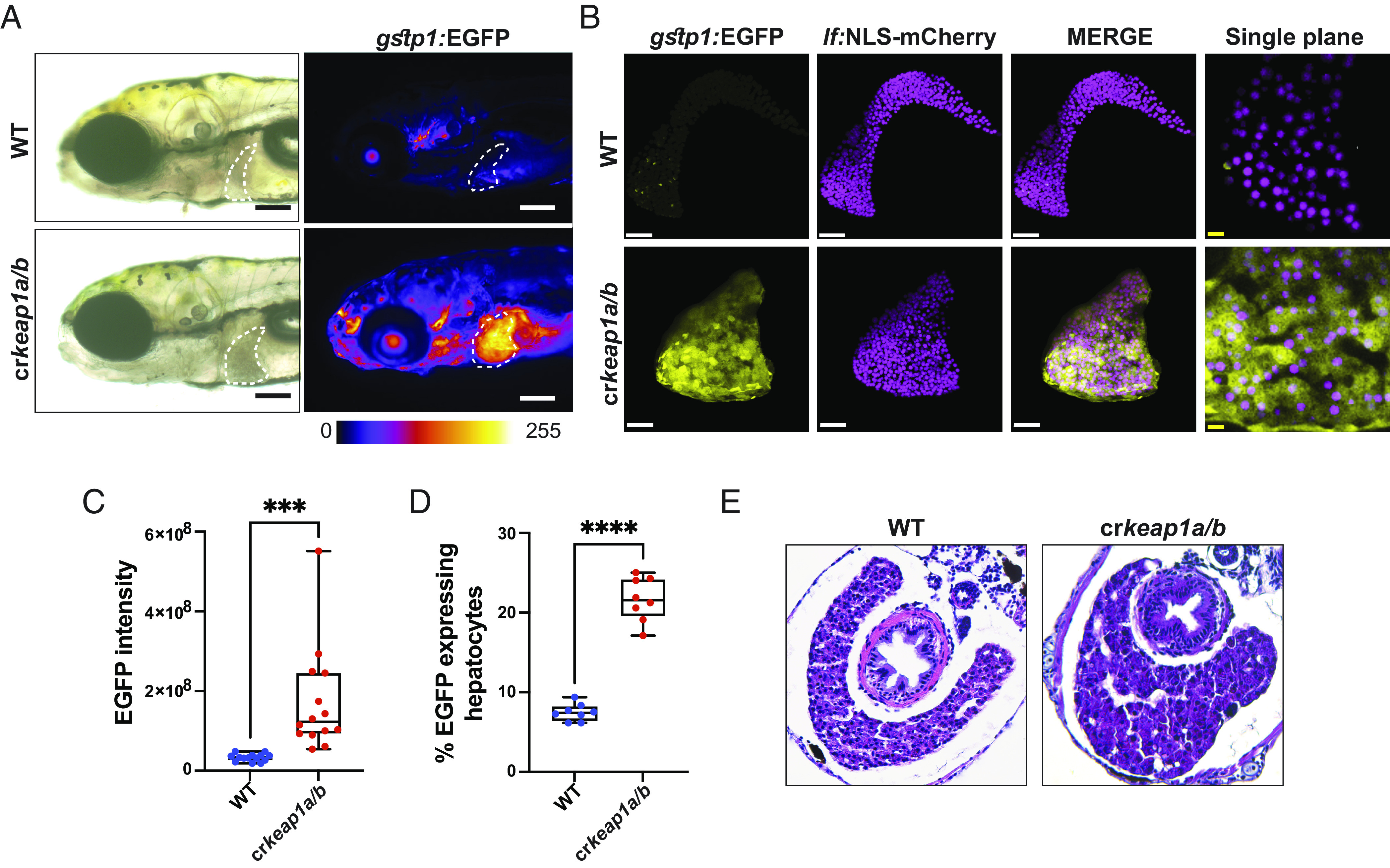
Keap1-deficient larvae exhibit defects in postembryonic liver development. (*A*) Representative whole-mount fluorescent images of WT and cr*keap1a/b* zebrafish on a *gstp1:*EGFP background at 7 dpf. Dashed line highlights the liver. Fluorescent images are pseudocolored using the Fire LUT. Scale bars represent 200 µm. (*B*) Representative Imaris-rendered multiphoton images of hepatocyte nuclei (magenta) and EGFP expression (yellow) in WT and cr*keap1a/b* zebrafish at 7 dpf. White scale bars represent 50 µm, yellow scale bars represent 10 µm. (*C*) Quantification of EGFP intensity in livers of WT and cr*keap1a/b* zebrafish at 7 dpf. Data are shown as mean and interquartile range, n = 14. (*D*) Quantification of EGFP expressing/mCherry-positive hepatocytes at 7 dpf as determined by flow cytometric analysis of larval single-cell suspensions, n= 8 pooled samples of 10 larvae. (*E*) Representative hematoxylin and eosin-stained transverse sections from WT and cr*keap1a/b* zebrafish at 7 dpf. For all experiments ***P < 0.001, ****P < 0.0001.

Additional analysis of cr*keap1a/b* zebrafish revealed Nrf2 reporter activity to be highest in the liver (Fig. 2*A*). In addition to increased Nrf2 activation, liver morphology was altered in cr*keap1a/b* larvae. Specifically, in contrast to the multilobed wild-type (WT) liver, the cr*keap1a/b* liver appeared as a single droplet-shaped lobe (Fig. 2*A*) Next, a hepatocyte reporter line (Tg(*lf*:NLS-mcherry)) was utilized to specifically examine Nrf2 activity in hepatocytes. While liver volume and hepatocyte number were unchanged in cr*keap1a/b* larvae, a dramatic increase in Nrf2 activity was observed in hepatocytes (Fig. 2 *B* and *C* and *SI Appendix*, Fig. S2 *A* and *B*). Flow cytometry analysis confirmed elevated Nrf2 reporter activity in hepatocytes isolated from cr*keap1a/b* larvae (Fig. 2*D*). Histological evaluation highlighted sinusoidal widening and increased size of hepatocyte nuclei (Fig. 2*E* and *SI Appendix*, Fig. S2*C*). Multiphoton analysis of cr*keap1a/b* larvae on a combined Nrf2 and vascular reporter background [Tg(*gstp1*:EGFP; *kdrl*:mCherry)] confirmed vasodilation (*SI Appendix*, Fig. S2*D*). Importantly, Nrf2 activity was not observed in the vasculature (*SI Appendix*, Fig. S2*D*). Collectively, these studies illustrate that hepatocytes are sensitive to loss of Keap1.

### Loss of Keap1 Induces Lysosomal Biogenesis.

**Fig. 3.**
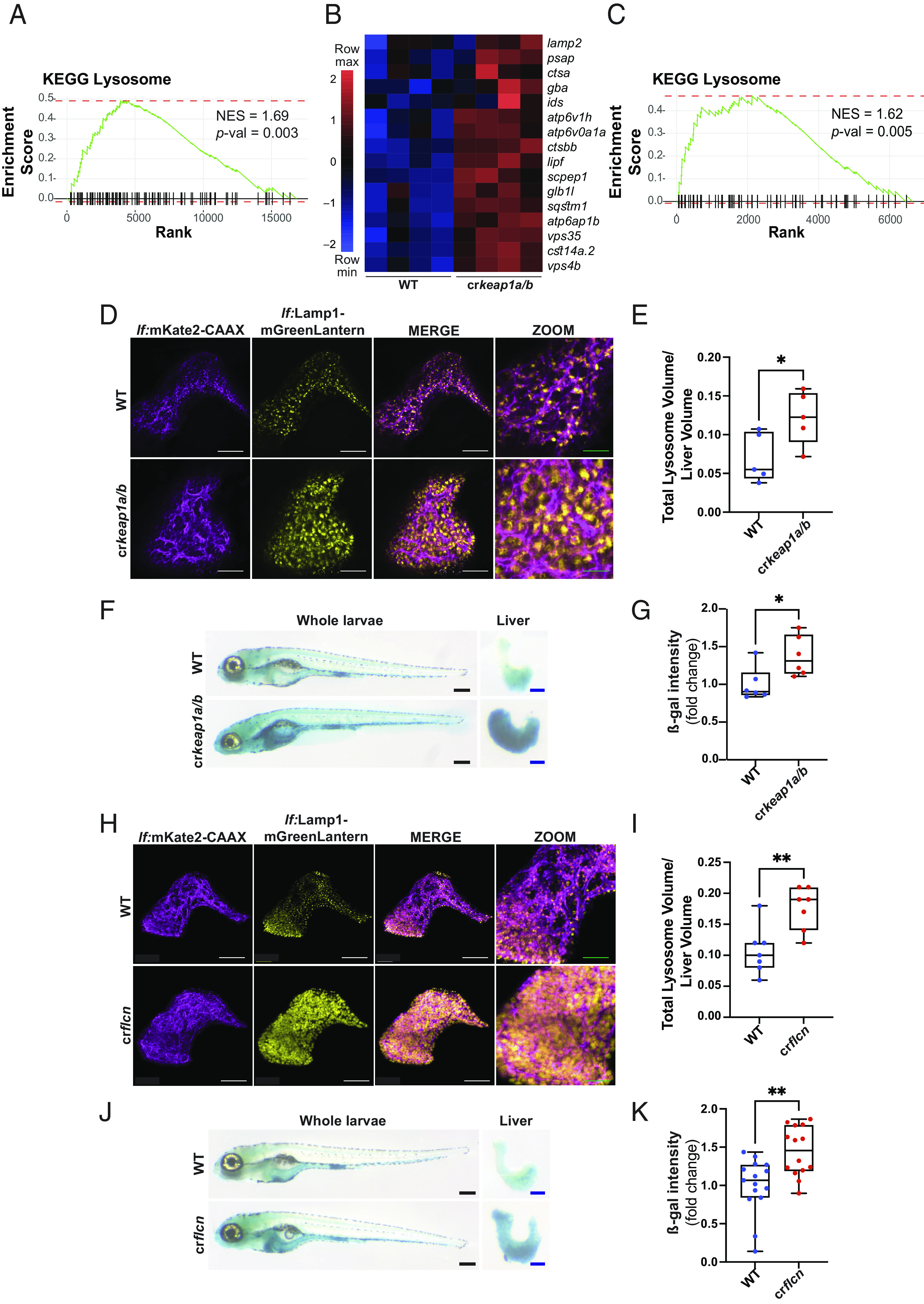
Loss of Keap1 induces lysosomal biogenesis. (*A*) GSEA plot, derived from RNA-Seq analysis of cr*keap1a/b* zebrafish versus their WT counterparts at 7 dpf, demonstrating induction of the KEGG lysosomal signature. (*B*) Heatmap of lysosomal genes among DEGs identified in Fig. 1*B*. (*C*) GSEA plot, derived from RNA-Seq analysis of dissected larval livers isolated from cr*keap1a/b* zebrafish and their WT counterparts at 7 dpf, demonstrating induction of the KEGG lysosomal signature, n = 3 pools of 15 larval livers. (*D*) Representative multiphoton images of hepatocyte membrane (magenta) and lysosomes (yellow) on a *lf:*Lamp1-mGreenLantern*; lf:*mKate2-CAAX background of WT and cr*keap1a/b* zebrafish at 7 dpf. White scale bars represent 50 µm, green scale bars represent 25 µm. (*E*) Quantification of lysosome volume (mGreenLantern) normalized to liver volume in *D*. (*F*) Representative images of ß-galactosidase (ß-gal)-stained whole-mount larvae (*Left*) and dissected larval livers (*Right*) of WT and cr*keap1a/b* zebrafish at 7 dpf. Black scale bars represent 350 µm, blue scale bars represent 100 µm. (*G*) Quantification of ß-gal intensity in dissected larval livers in *F*. (*H*) Representative multiphoton images of hepatocyte membrane (magenta) and lysosomes (yellow) on a *lf:*Lamp1-mGreenLantern*; lf:*mKate2-CAAX background of WT and cr*flcn* zebrafish at 7 dpf. White scale bars represent 50 µm, green scale bars represent 25 µm. (*I*) Quantification of lysosome volume (mGreenLantern) normalized to liver volume in *H*. (*J*) Representative images of ß-gal-stained whole-mount larvae (*Left*) and dissected larval livers (*Right*) of WT and cr*flcn* zebrafish at 7 dpf. Black scale bars represent 350 µm, blue scale bars represent 100 µm. (*K*) Quantification of of ß-gal intensity in dissected larval livers in *J*. For all quantification, data are shown as mean and interquartile range, *P < 0.05, **P < 0.01.

Among the most enriched pathways observed by GSEA in cr*keap1a/b* larvae was the KEGG lysosomal signature (Fig. 3*A*). Indeed, expression of numerous lysosomal genes, including ß-galactosidase (ß-gal) and multiple cathepsins, was notably increased in the context of Keap1 loss (Fig. 3*B*). The lysosomal signature was also highly enriched in dissected larval liver tissue (Fig. 3*C*). Transmission electron microscopy of cr*keap1a/b* livers, relative to WT livers, revealed an increase in single membrane-bound vesicles, suggesting an increase in lysosomal abundance (*SI Appendix*, Fig. S3*A*). To directly visualize lysosomes in the liver, a hepatocyte-specific lysosomal reporter [Tg(*lf*:Lamp1-mGreenLantern; *lf*:mKate2-CAAX)] was generated. In this line, a dramatic increase in lysosome abundance was observed in response to loss of Keap1 (Fig. 3 *D* and *E*). To visualize lysosomal activity in the whole larvae, ß-gal staining was performed. In contrast to WT larvae, which exhibited ß-gal staining in lysosome-rich enterocytes (21), cr*keap1a/b* larvae displayed intense ß-gal staining in the liver (Fig. 3 *F* and *G*). Although ß-gal staining is used as a measure of lysosomal activity (22), it is also used as a surrogate marker of cellular senescence (23). Therefore, orthogonal assays were employed to exclude a role for senescence in the Keap1-deficient phenotype. A characteristic feature of senescence is p53-dependent cell cycle arrest (24, 25)(22). Co-staining of ß-gal and proliferating cell nuclear antigen in cr*keap1a/b* hepatocytes revealed no evidence of cell cycle arrest (*SI Appendix*, Fig. S3*B*). Moreover, loss of p53 did not impact ß-gal staining (*SI Appendix*, Fig. S3 *C* and *D*). Further analysis of the RNA-Seq datasets revealed an absence of senescence-associated secretory phenotype factors among the DEGs observed in cr*keap1a/b* larval livers and whole larvae (*SI Appendix*, Fig. S3 *E* and *F*). These data suggest that increased lysosomal biogenesis occurs as a consequence of Keap1 loss.

Expression of lysosomal genes, and regulation of lysosomal biogenesis, is controlled by members of the MiT/TFE family of transcription factors, which include TFEB and TFE3 (26). Loss of folliculin (FLCN) stimulates lysosomal biogenesis by promoting TFEB/TFE3 activation (27, 28)(26). A CRISPR/Cas9 gene editing approach was employed to compare the developmental phenotypes observed following loss of Keap1 or loss of Flcn. Mosaic KO of *flcn* (cr*flcn*) on the hepatocyte-specific lysosomal reporter background phenocopied the increase in lysosome abundance and activity observed in cr*keap1a/b* larvae (Fig. 3 *H*–*K* and *SI Appendix*, Fig. S3 *G*–*I*). Interestingly, loss of Flcn or Keap1 was associated with mortality at a similar developmental stage, suggesting that dysregulation of lysosomal biogenesis may contribute to postembryonic lethality (*SI Appendix*, Fig. S3*J*).

### Bach1-Mediated Repression of Nrf2 Modulates Lysosomal Biogenesis.

**Fig. 4.**
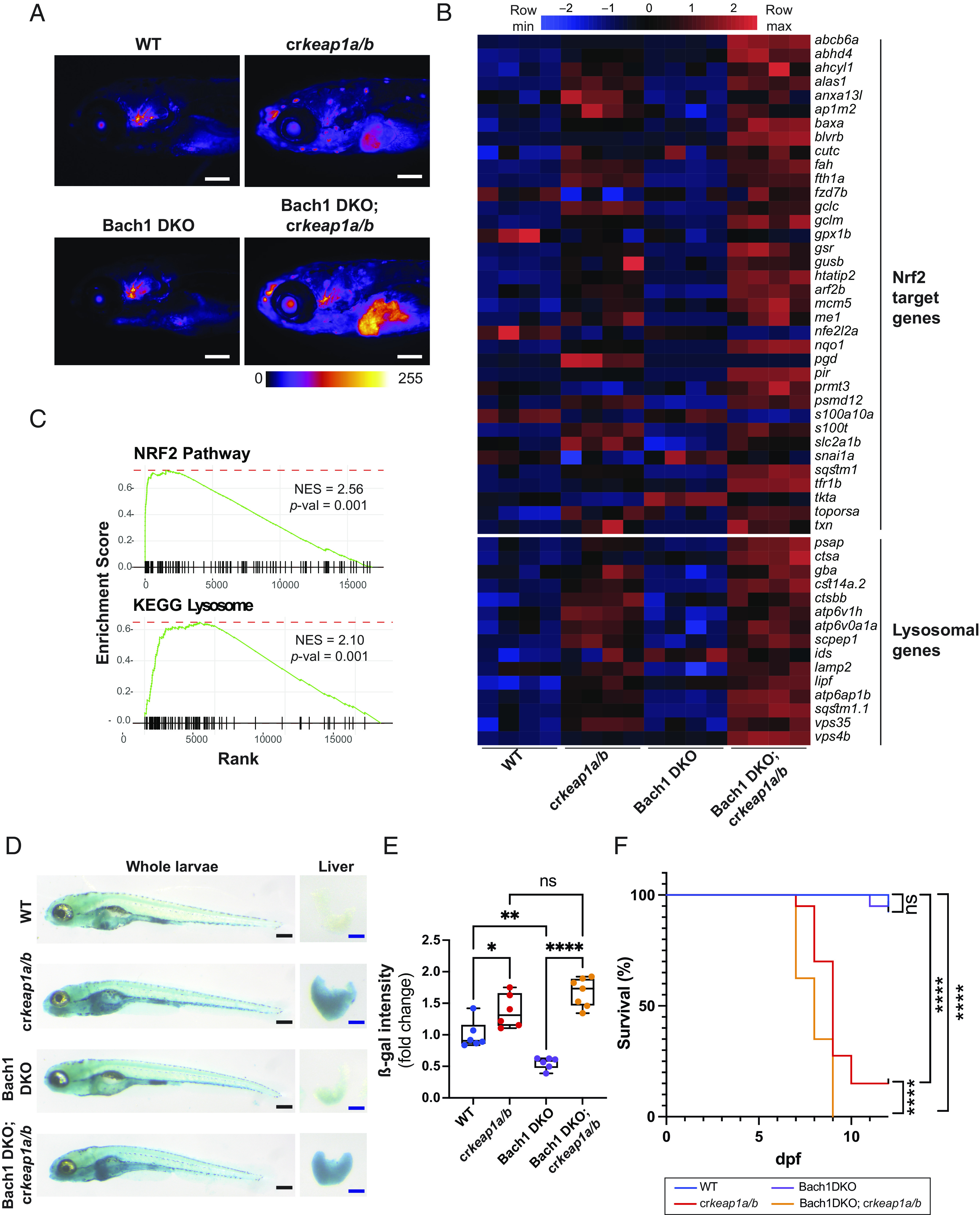
Bach1-mediated repression of Nrf2 modulates lysosomal biogenesis. (*A*) Representative whole-mount fluorescent images of WT, cr*keap1a/b*, Bach1 DKO, and Bach1 DKO; cr*keap1a/b* zebrafish on a *gstp1:*EGFP background at 7 dpf. Fluorescent images are pseudocolored using the Fire LUT. Scale bars represent 200 µm. (*B*) Heatmap of Nrf2 target genes and lysosomal genes in WT, cr*keap1a/b*, Bach1 DKO, and Bach1 DKO; cr*keap1a/b* zebrafish at 7 dpf as determined by RNA-Seq analysis, n = 4 pools of 10 larvae. (*C*) GSEA plots derived from RNA-Seq analysis of Bach1 DKO; cr*keap1a/b* versus Bach1 DKO zebrafish at 7 dpf demonstrating the Nrf2 pathway signature and KEGG lysosomal signature. (*D*) Representative images of ß-gal-stained whole-mount (*Left*) and dissected larval livers (*Right*) from WT, cr*keap1a/b*, Bach1 DKO, and Bach1 DKO; cr*keap1a/b* zebrafish at 7 dpf. Black scale bars represent 350 µm, blue scale bars represent 100 µm. (*E*) Quantification of ß-gal intensity in dissected larval livers represented in *D*. Data are shown as mean and interquartile range, n = 6 to 7. (*F*) Kaplan–Meier survival plot of WT, cr*keap1a/b*, Bach1 DKO, and Bach1 DKO; cr*keap1a/b* zebrafish, n = 40. For all experiments, *P < 0.05, **P < 0.01, ****P < 0.0001.

In addition to enrichment of genes regulated by NRF2, HOMER de novo motif analysis identified BTB domain and CNC homolog 1 (BACH1) as one of the most enriched motifs in cr*keap1a/b* larvae (Fig. 1*C*). BACH1 is a transcriptional repressor that regulates heme metabolism via binding to MAF recognition elements (MARE). Due to the similarity of MARE and ARE motifs, a subset of NRF2 target genes are known to be co-regulated by BACH1 (29, 30). To explore the role of BACH1 in KEAP1-regulated lysosomal biogenesis, Bach1 double KO (Bach1 DKO) zebrafish were generated on a Tg(*gstp1*:EGFP; *lf*:NLS-mcherry) background (*SI Appendix*, Fig. S4*A*). Loss of Bach1 had no effect on Nrf2 reporter activity in the WT background (Fig. 4*A*). RNA-Seq analysis confirmed that Bach1 loss was not sufficient to induce the NRF2 transcriptional program (Fig. 4*B*). However, in the context of Keap1 deficiency, loss of Bach1 exacerbated Nrf2 reporter activity (Fig. 4*A*), and this was reflected by augmented NRF2 target gene expression (Fig. 4 *B* and *C*). Moreover, GSEA revealed a higher normalized enrichment score for multiple features, including the KEGG lysosome signature, in Bach1 DKO; cr*keap1a/b* compound mutants relative to cr*keap1a/*b mutant larvae (Figs. 1*E*, 3*A*, and 4*C* and *SI Appendix*, Fig. S4*B*). ß-gal staining confirmed a significant increase in lysosomal biogenesis in Bach1 DKO; cr*keap1a/b* larvae (Fig. 4 *D* and *E*). Importantly, loss of Bach1 accelerated Keap1-dependent postembryonic lethality (Fig. 4*F*). Collectively these results suggest that, during embryonic development, BACH1 contributes to repression of NRF2 activity.

### Keap1-Dependent Regulation of Lysosomal Biogenesis Requires Nrf2.

**Fig. 5.**
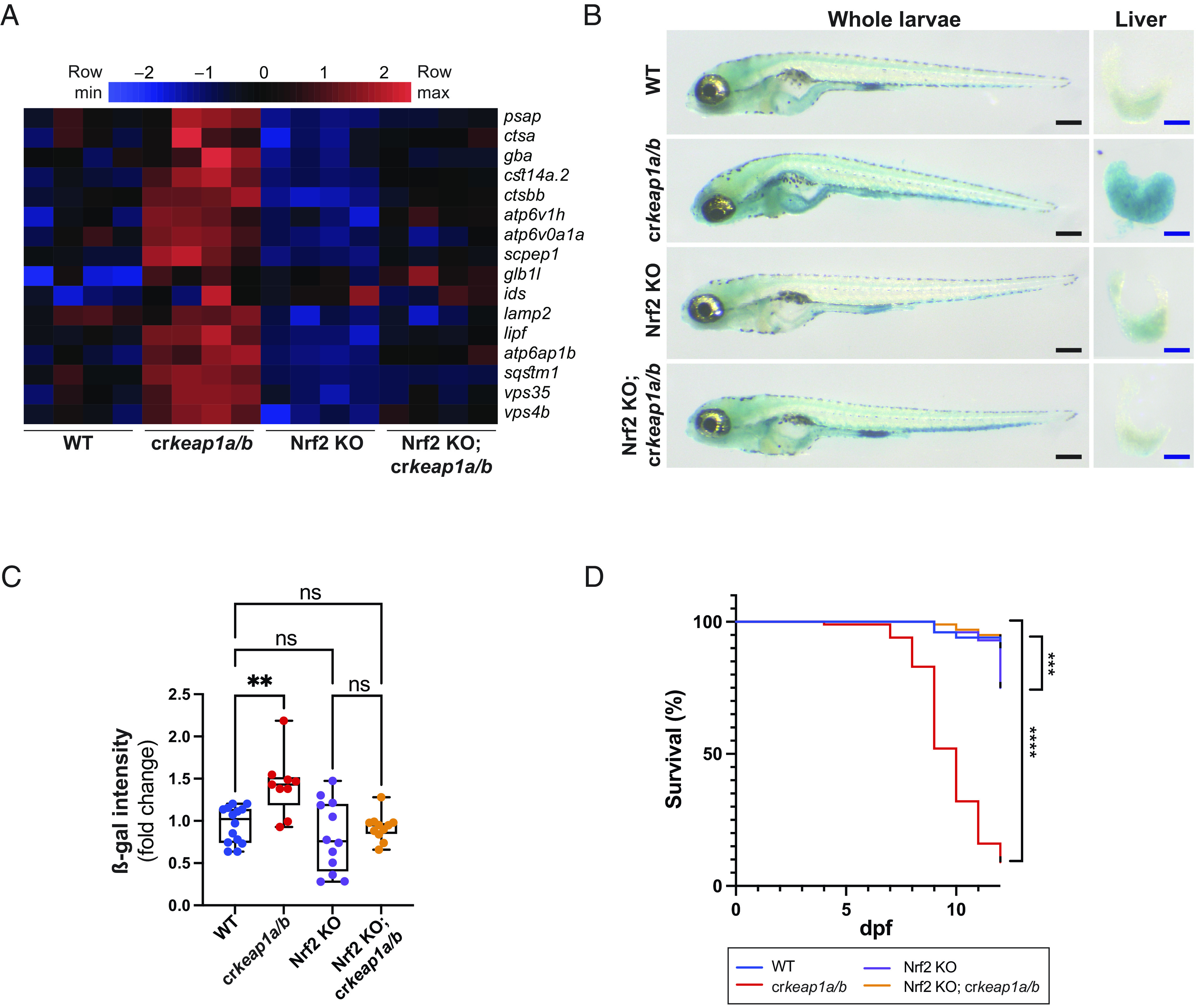
Keap1-dependent regulation of lysosomal biogenesis requires Nrf2. (*A*) Heatmap of lysosomal gene expression in WT, cr*keap1a/b*, Nrf2 KO, and Nrf2 KO; cr*keap1a/b* zebrafish at 7 dpf as determined by RNA-Seq analysis, n = 4 pools of 10 larvae. (*B*) Representative images of ß-gal-stained whole-mount larvae (*Left*) and dissected larval livers (*Right*) from WT, cr*keap1a/b*, Nrf2 KO, and Nrf2 KO; cr*keap1a/b* zebrafish at 7 dpf. Black scale bars represent 350 µm, blue scale bars represent 100 µm. (*C*) Quantification of ß-gal intensity in dissected livers in *B*. Data are shown as mean and interquartile range, n = 9 to 14 livers. (*D*) Kaplan–Meier survival plot of WT, cr*keap1a/b*, Nrf2 KO, and Nrf2 KO; cr*keap1a/b* zebrafish, n = 40. For all experiments, **P < 0.01, ***P < 0.001, ****P < 0.0001.

To determine whether NRF2 is required for lysosomal biogenesis in the context of KEAP1 deficiency, we took advantage of a previously reported Nrf2 mutant line (*nfe2l2*^fh318^) (16, 31). Keap1 loss failed to induce the NRF2 transcriptional program in the Nrf2 mutant background (*SI Appendix*, Fig. S5*A*). In addition, the increase in lysosomal gene expression observed in cr*keap1a/b* larvae was suppressed by loss of Nrf2 activity (Fig. 5*A*). Moreover, the increase in ß-gal staining and postembryonic lethality observed in cr*keap1a/b* larvae were rescued in the Nrf2 mutant background (Fig. 5 *B*–*D*). These results demonstrate that KEAP1-dependent lysosomal biogenesis and postembryonic lethality are dependent on NRF2.

### KEAP1-Dependent Regulation of Lysosomal Biogenesis Is Cell-Autonomous and Evolutionarily Conserved.

**Fig. 6.**
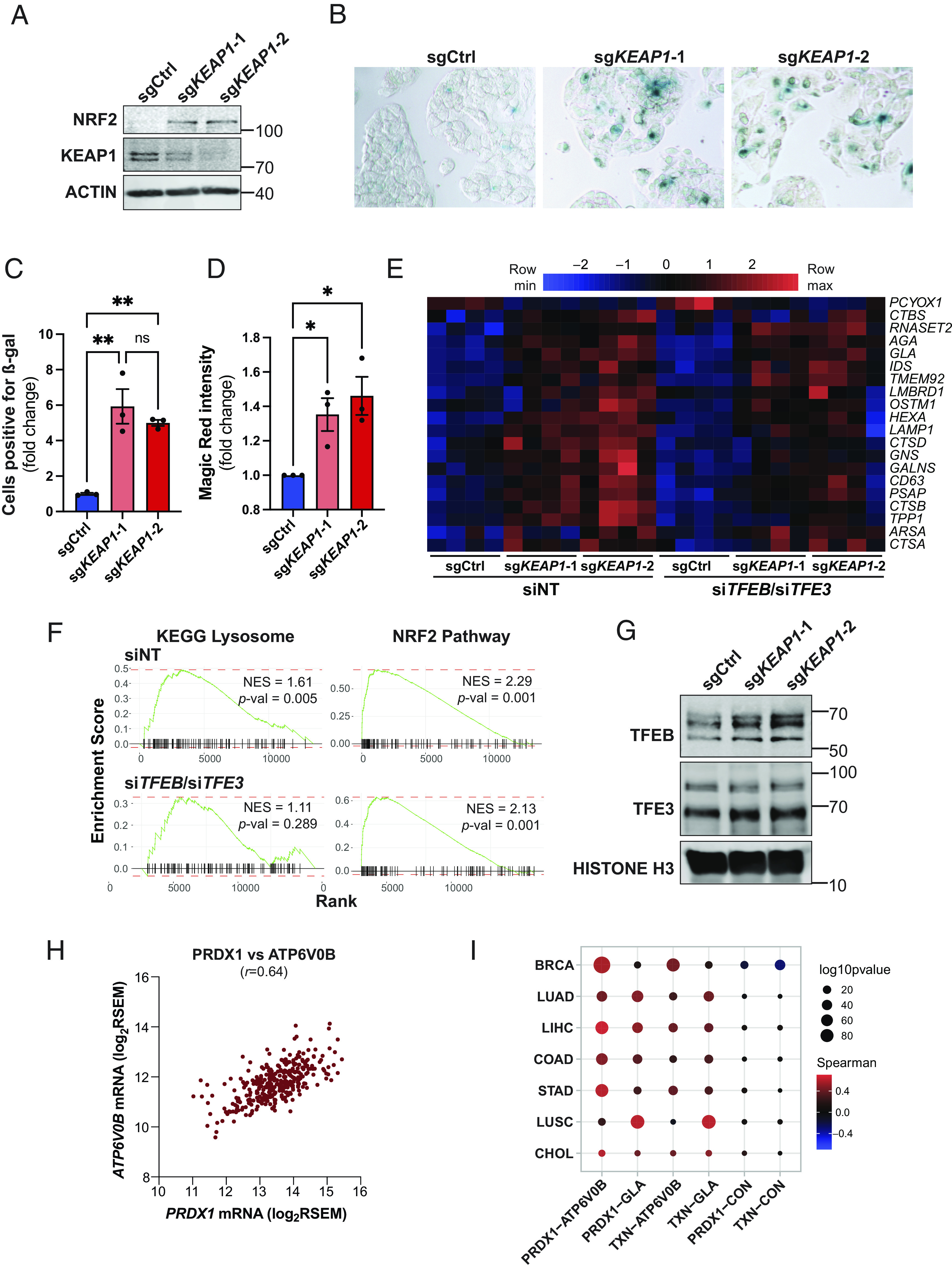
KEAP1-dependent regulation of lysosomal biogenesis is cell-autonomous and evolutionarily conserved. (*A*) Representative immunoblot analysis of HepG2 cells transduced with Cas9/guide RNA constructs targeting *AAVS1* (sgCtrl) and *KEAP1* (sg*KEAP1*-1, sg*KEAP1*-2). (*B*) Representative images of ß-gal-stained sgCtrl, sg*KEAP1*-1, and sg*KEAP1*-2 HepG2 cells. (*C*) Quantification of ß-gal-positive cells in sg*KEAP1*-1, and sg*KEAP1*-2 HepG2 cells relative to sgCtrl cells. Data are shown as mean ± SEM, n = 3. (*D*) Quantification of Magic Red fluorescence intensity in sg*KEAP1*-1, and sg*KEAP1*-2 HepG2 cells relative to sgCtrl cells. Data are shown as mean ± SEM, n = 3. (*E*) Heatmap of lysosomal gene expression in sgCtrl, sg*KEAP1*-1, and sg*KEAP1*-2 HepG2 cells transfected with either non-targeting siRNAs (siNT) or siRNAs targeting *TFEB* and *TFE3* (si*TFEB*/si*TFE3*) as determined by RNA-Seq analysis, n = 4. (*F*) GSEA plots derived from RNA-Seq analysis of sg*KEAP1*-2 versus sgCtrl cells transfected with either siNT or si*TFEB*/si*TFE3* demonstrating the KEGG lysosome pathway and Nrf2 pathway signatures. (*G*) Representative immunoblot analysis of nuclear fractions isolated from sgCtrl, sg*KEAP1*-1, and sg*KEAP1*-2 HepG2 cells. (*H*) Dot plot showing a correlation between *PRDX1* and *ATP6V0B* mRNA expression in the TCGA Liver Hepatocellular Carcinoma (LIHC) dataset. (*I*) Spearman’s rank-order correlation of expression of the Nrf2 target genes *PRDX1* and *TXN* with the lysosomal genes *ATP6V0B* and *GLA* in TCGA datasets.

To determine whether regulation of lysosomal biogenesis by KEAP1 occurs in a cell-autonomous and evolutionarily conserved manner, the impact of KEAP1 loss was also examined using mammalian cells. Given that KEAP1 loss primarily impacted the liver in vivo (Fig. 2), a hepatic cell line (HepG2) was employed. KEAP1 KO HepG2 cells were generated using a CRISPR/Cas9 gene editing approach. Consistent with the in vivo data, loss of KEAP1 induced a marked increase in NRF2 expression and GSH content (Fig. 6*A* and *SI Appendix*, Fig. S6*A*). Moreover, loss of KEAP1 was associated with a significant increase in ß-gal staining (Fig. 6 *B* and *C*). KEAP1 KO HepG2 cells also exhibited elevated cathepsin activity and increased lysosomal abundance, as determined using the Magic Red cathepsin activity assay and the acidotropic dye LysoTracker, respectively (Fig. 6*D* and *SI Appendix*, Fig. S6*B*). To examine the role of TFEB/TFE3 in KEAP1 KO cells, RNA-Seq analysis was performed in the absence or presence of siRNA-mediated knockdown of TFEB and TFE3 (*SI Appendix*, Fig. S6*C*). NRF2 target genes and lysosomal genes were up-regulated in KEAP1 KO HepG2 cells (Fig. 6*E* and *SI Appendix*, Fig. S6*C*). Moreover, enrichment of the NRF2 pathway signature and the KEGG lysosome signature was revealed by GSEA (Fig. 6*F*). Importantly, TFEB/TFE3 knockdown suppressed lysosomal gene expression in KEAP1 KO HepG2 cells without any impact on NRF2 target gene expression (Fig. 6 *E* and *F* and *SI Appendix*, Fig. S6*D*). Consistent with increased lysosomal gene expression, elevated nuclear localization of TFEB/TFE3 was observed in KEAP1 KO cells (Fig. 6*G*). Analysis of gene expression data from the The Cancer Genome Atlas database revealed a strong correlation between expression of the NRF2 target genes *PRDX1 and TXN* and expression of the TFEB/TFE3 target genes *ATP6V0B and GLA* (Fig. 6 *H* and *I* and *SI Appendix*, Fig. S6*E*). Collectively, these data indicate that KEAP1 loss induces a TFEB/TFE3-dependent lysosomal program in a cell-autonomous and evolutionarily conserved manner.

## Discussion

The NRF2 pathway is recognized as an important regulator of redox homeostasis and cellular metabolism. There is clear evidence that NRF2 can regulate both anabolic (GSH biosynthesis, pentose phosphate pathway, and serine biosynthesis) and catabolic processes [autophagy, heme catabolism, and fatty acid oxidation (FAO)] (32–34), although in some cases, the mechanisms involved are poorly understood. Many of the mechanistic insights into the regulation of metabolism by NRF2 have relied on cancer models harboring confounding oncogenic mutations (13, 35, 36). In these circumstances, there may be aspects of NRF2 biology that are more difficult to detect. Here, we have examined the function of NRF2 in a nontumorigenic background. Using this approach, we reveal an evolutionarily conserved mechanism wherein the KEAP1-NRF2 pathway regulates TFEB/TFE3-dependent lysosomal biogenesis.

Lysosomes have traditionally been viewed as hubs of catabolic activity, harboring a plethora of proteases and hydrolases involved in degrading a range of macromolecules (37). More recently, lysosomes have been recognized to play a central role in nutrient sensing in part due to the lysosomal localization of the active mTORC1 complex (38). At the transcriptional level, lysosomal biogenesis is regulated by the MiT/TFE family of transcription factors, which includes TFEB and TFE3 (39–41). TFEB and TFE3 localization and activity are linked to nutrient availability via mTORC1-dependent phosphorylation (42, 43). The emerging consensus is that lysosomal abundance and activity are regulated by switching between the anabolic mTORC1 signaling cascade (44) and catabolic TFEB/TFE3 network (39–41). In this study, we have demonstrated that KEAP1 loss drives an NRF2-dependent increase in lysosomal biogenesis. This is consistent with a recent study demonstrating an increase in lysosomal content in Keap1 KO mouse embryonic fibroblasts (45). Another study has shown that loss of NRF2 decreases TFEB nuclear localization and TFEB-dependent transcription of the lysosomal protein VAMP8, which supports a role for NRF2 in the regulation of TFEB (46). It would be of interest in subsequent studies to determine whether the NRF2 pathway plays a buffering role in the regulation of lysosomal homeostasis in disease. For instance, aberrant proteostasis and defective lysosomal biogenesis can drive the pathogenesis of a subset of neurodegenerative diseases (47–49). There may be opportunities to examine the efficacy of NRF2 activating compounds as a therapeutic approach to treat these disorders.

Previous work supports the notion that lysosomal biogenesis is sensitive to changes in redox state (50–52). In the current study, we found that loss of Keap1 promotes accumulation of GSH during embryonic development. Furthermore, four NRF2 target genes (*G6PD*, *PGD*, *ME1*, and *IDH1*) generate NAPDH. Elevated levels of reducing equivalents, such as GSH and NADPH, can promote reductive stress (53). Reductive stress has been shown to impair processes important for development, including myogenic differentiation (53) and neurogenesis (54). Until recently, little was known about the molecular underpinnings of how cells sense reductive stress. However, recent insights have shed light on this question by identifying folliculin-interacting protein 1 (FNIP1) as a sensor of reductive stress (53, 55). Interestingly, FNIP1 forms a heterodimeric complex with FLCN on the cytoplasmic surface of lysosomes (28), which acts as a GTPase-activating protein to stimulate mTORC1 and inhibit TFEB/TFE3 (56).

The regulation of FAO by NRF2 has been controversial in the field, in part, because the genes involved in FAO do not harbor NRF2-binding sites (AREs) in their promoter regions. Interestingly, TFEB and TFE3 have been shown to regulate the transcription of genes involved in FAO in vivo (57, 58). This may provide a mechanistic link that explains how NRF2 can regulate FAO. In a similar vein, it is now well established that NRF2 can regulate autophagy in the context of cancer (59)(14). Given that TFEB/TFE3 induce genes involved in the execution of autophagy (60), it would be interesting to examine the requirement for TFEB/TFE3 in situations where NRF2 induces autophagy. Together, this body of research suggests that TFEB/TFE3 may play a critical role in facilitating NRF2-driven catabolic processes.

BACH1 has historically been shown to act as a transcriptional repressor, suppressing expression of NRF2 target genes involved in the antioxidant response (61–63). However, recent studies in lung cancer models (harboring Kras and p53 mutations) have shown that BACH1 is required for metastasis downstream of NRF2 activation (64, 65). In the context of embryonic development, we found that Bach1 represses Nrf2 activity in Keap1-deficient larvae, but not basal Nrf2 activity in WT larvae. Clearly, context must play a key role in determining the repressive functions of BACH1 (66). It is also possible that downstream functions of BACH1 are tissue-dependent in part due to differences in heme abundance (67, 68).

In conclusion, this study has revealed that KEAP1 loss promotes lysosomal biogenesis. Mechanistically, we provide evidence that NRF2 activates a conserved TFEB/TFE3-dependent program. Given that KEAP1 and NRF2 are frequently mutated in cancer (69), it will be interesting to explore the involvement of TFEB/TFE3 in NRF2-driven cancers.

## Materials and Methods

### Zebrafish Husbandry.

Zebrafish were maintained according to institutional Animal Experimentation and Ethics Committee (AECC) guidelines (Approval E580, E666, E634). Previously described lines used in this study: Tg(*gstp1:*EGFP) (70), Tg(*fabp10a:*NLS-mCherry*)* (71), p53 KO (*tp53*^M214K^) (72), Nrf2 KO (*nfe2l2*^fh318^) (16), Tg(*kdrl:*HsHRAS-mCherry)^S916^ (73). Transgenic lines generated in this study: Tg(*lf*:Lamp1-mGreenLantern)^uom302^ and Tg(*lf*:mKate2-CAAX)^uom303^. Compound *bach1a/bach1b* mutants, herein referred to as Bach1 DKO^uom301^, were generated by crossing cr*bach1a* (bach1a^R49*, S93*^) and cr*bach1b* (bach1b^S16*^) mutants generated using the CRISPR/Cas9 method described below. For all experiments, clutch siblings were used as controls and all embryos and larvae were maintained at 28 °C throughout development. All zebrafish experiments were performed at the larval stage and therefore the sex of the organism was not yet determined. All other zebrafish experimental methods are provided in *SI Appendix*.

### Cell Culture.

HepG2 cells were purchased from CellBank Australia and maintained in MEM with NEAA (ThermoFisher Scientific, 10370) supplemented with 1 mM sodium pyruvate, 2 mM glutamine, and 10% fetal bovine serum. In addition, 293T cells were acquired from American Type Culture Collection and maintained in Dulbecco's Modified Eagle Medium (ThermoFisher Scientific, 11965) supplemented with 1 mM sodium pyruvate and 10% FBS. Cells were maintained a humidified incubator at 37 °C with 5% CO_2_. Cell lines were authenticated using short tandem repeat profiling and routinely assayed for *mycoplasma* contamination. All other cell culture experimental methods are provided in *SI Appendix*.

### Quantification and Statistical Analysis.

Statistical analyses were performed with Prism 9 software (GraphPad Software). All statistical analyses for data comparing two groups were performed with an unpaired Student’s *t* test. One-way ANOVA with the Holm–Sidak method for multiple comparisons was used for comparison of more than two groups. Survival curves were compared pairwise and the statistical significance was determined using the Gehan–Breslow–Wilcoxon method. All immunoblots are representative of results from at least three independent experiments. All other statistical details of experiments can be found in the figure legends.

## Supplementary Material

Appendix 01 (PDF)

## Data Availability

The RNA-seq data reported in this paper have been deposited in the GEO database (accession no. GSE230611) (74). All study data are included in the article and/or *SI Appendix*.
