# Supplementary material for: The KEAP1–NRF2 pathway regulates TFEB/TFE3-dependent lysosomal biogenesis
Source: Proc Natl Acad Sci U S A. 2023 May 22;120(22):e2217425120. doi: 10.1073/pnas.2217425120 (PMC10235939; doi:10.1073/pnas.2217425120)
Supplement: Supplementary file 1 — Appendix 01 (PDF) [file pnas.2217425120.sapp.pdf]

Supplementary Figures

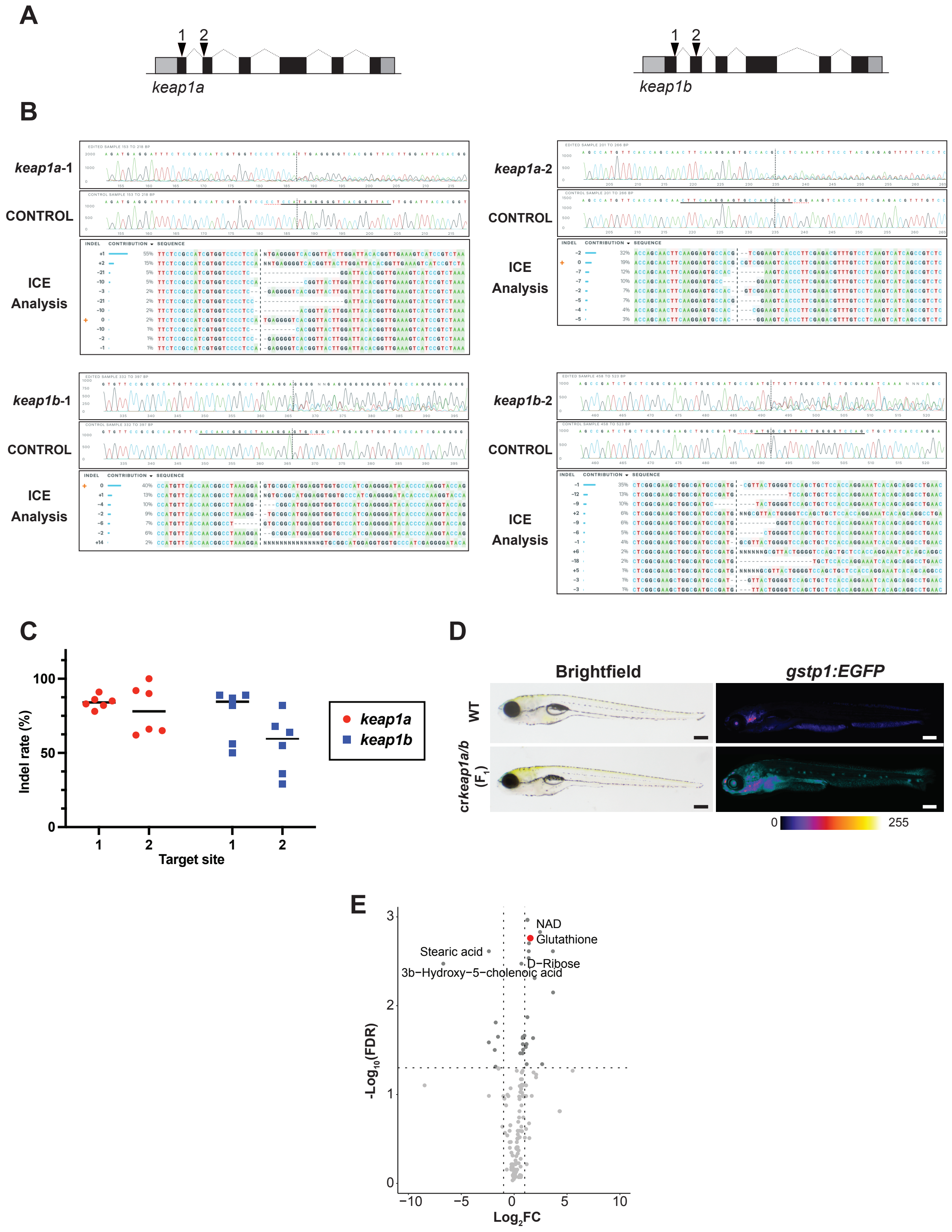

Fig. S1. Loss of Keap1 activates Nrf2 and drives postembryonic lethality.

(A) Schematic of CRISPR guide target sites in *keap1a* and *keap1b*. (B) Sanger sequencing and ICE analysis of CRISPR-induced indels in zebrafish larvae at 7 dpf injected with 4 guides targeting *keap1a* (sites 1 and 2) and *keap1b* (sites 1 and 2) at the 1-cell stage. (C) Indel rates of the CRISPR target sites on *keap1a* and *keap1b,* n=6 larvae. (D) Representative whole-mount brightfield and fluorescent images of WT and first filial generation (F_1) cr*keap1a/b* zebrafish on a *gstp1:*EGFP background at 7 dpf. Fluorescent images are pseudo-coloured using a pseudo-coloured Fire LUT. Scale bars represent 350 µm. (E) Volcano plot depicting the 165 polar metabolites detected in WT and cr*keap1a/b* larvae at 7 dpf, n=4 pools of 10 larvae. Metabolites significantly suppressed or enriched in cr*keap1a/b* larvae are highlighted in dark grey. GSH is highlighted in red.

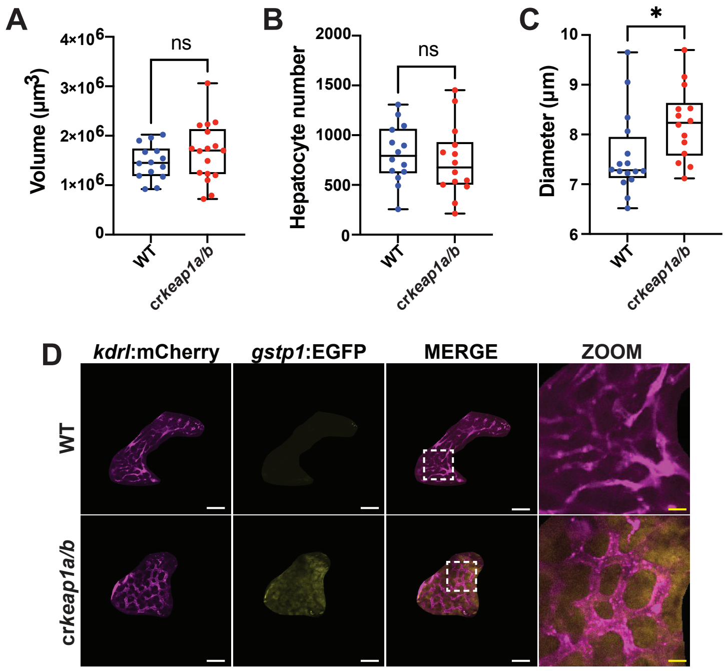

Fig. S2. Keap1 deficient larvae exhibit defects in postembryonic liver development.

Quantification of (A) liver volume, (B) hepatocyte number, and (C) hepatocyte nuclei diameter in WT and cr*keap1a/b* zebrafish at 7 dpf as determined by multiphoton microscopy. Data are shown as mean and interquartile range. (D) Representative multiphoton images of liver vasculature in WT and cr*keap1a/b* zebrafish on a *gstp1*:EGFP; *kdrl*:mCherry background at 7 dpf. White scale bars represent 50 µm. Yellow scale bars represent 10 µm. For all experiments *P<0.05, ns is not significant.

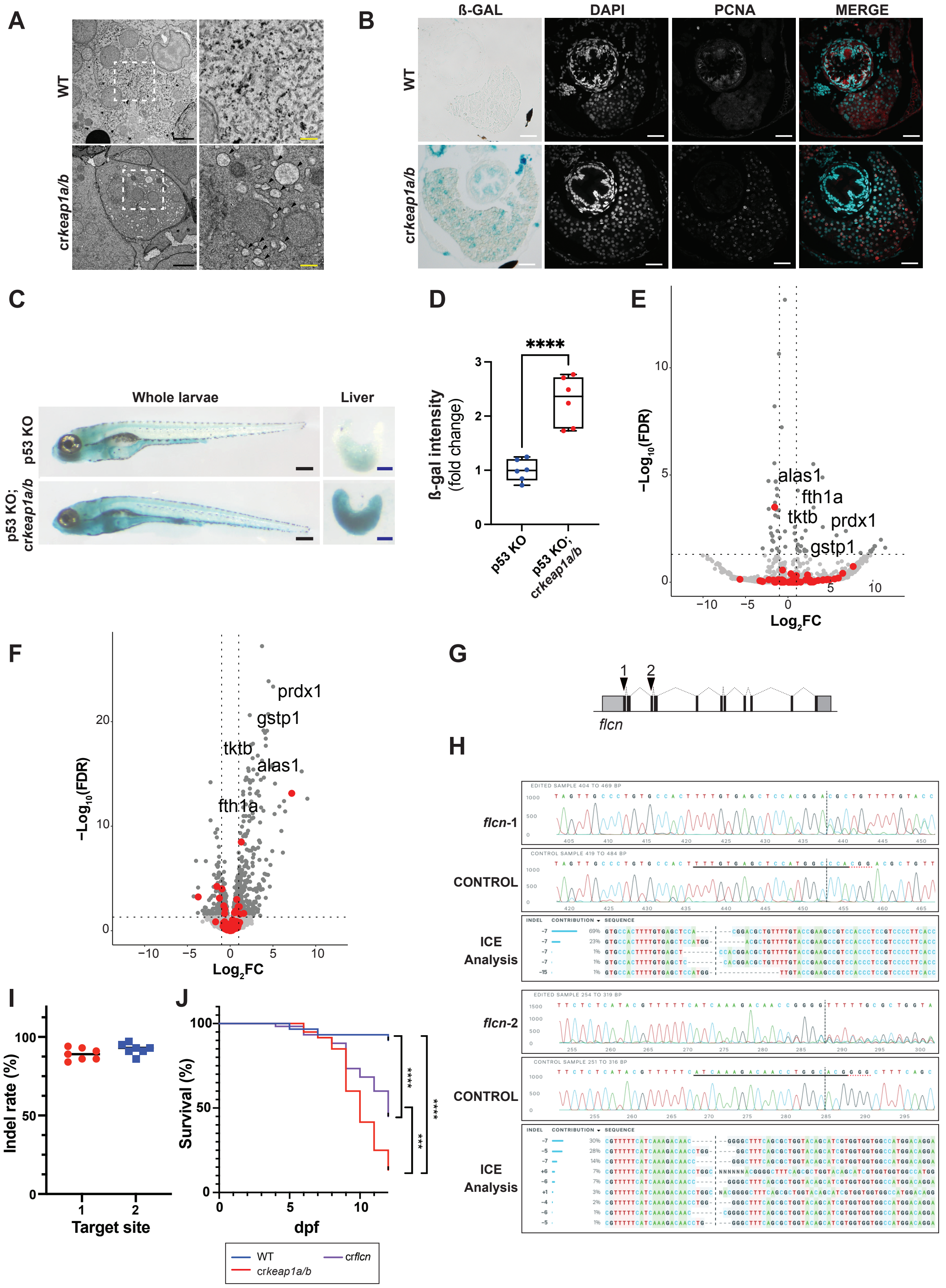

Fig. S3. Loss of Keap1 induces lysosomal biogenesis.

(A) Representative transmission electron microscopy (TEM) images of liver sections from WT and cr*keap1a/b* zebrafish at 7 dpf. Black arrows indicate single membrane-bound vesicles. Black scale bars represent 2 µm, yellow scale bars represent 500 nm. (B) Representative staining of ß-gal, DAPI and

PCNA in transverse larval liver sections from cr*keap1a/b* zebrafish and their WT counterparts at 7 dpf. Scale bars represent 30 µm. (C) Representative images of ß-gal stained whole-mount larvae (left) and dissected larval livers (right) from p53 KO and p53 KO; cr*keap1a/b* zebrafish at 7 dpf. Black scale bars represent 350 µm, blue scale bars represent 100 µm. (D) Quantification of ß-gal staining intensity in dissected larval livers represented in (C). Data are shown as mean and interquartile range, n=6. (E) Volcano plots of DEGs identified by RNA-Seq analysis comparing dissected larval livers from cr*keap1a/b* zebrafish and their WT counterparts at 7 dpf, n=3 pools of 15 dissected livers. Significantly DEGs are highlighted in dark grey. Senescence associated secretory phenotype (SASP) genes are highlighted in red. (F) Volcano plot of DEGs identified by RNA-Seq analysis of cr*keap1a/b* zebrafish and their WT counterparts at 7 dpf. Significantly DEGs are highlighted in dark grey. SASP genes are highlighted in red. (G) Schematic of CRISPR guide target sites in *flcn*. (H) Sanger sequencing and ICE analysis of CRISPR-induced indels in zebrafish larvae at 7 dpf injected with 2 guides targeting *flcn* (sites 1 and 2) at the 1-cell stage. (I) Indel rates of the CRISPR target sites on *flcn*. n=6 larvae. (J) Kaplan-Meier survival plot of WT, cr*keap1a/b*, and cr*flcn* zebrafish, n=60. For all experiments, ***P < 0.001, ****P < 0.0001.

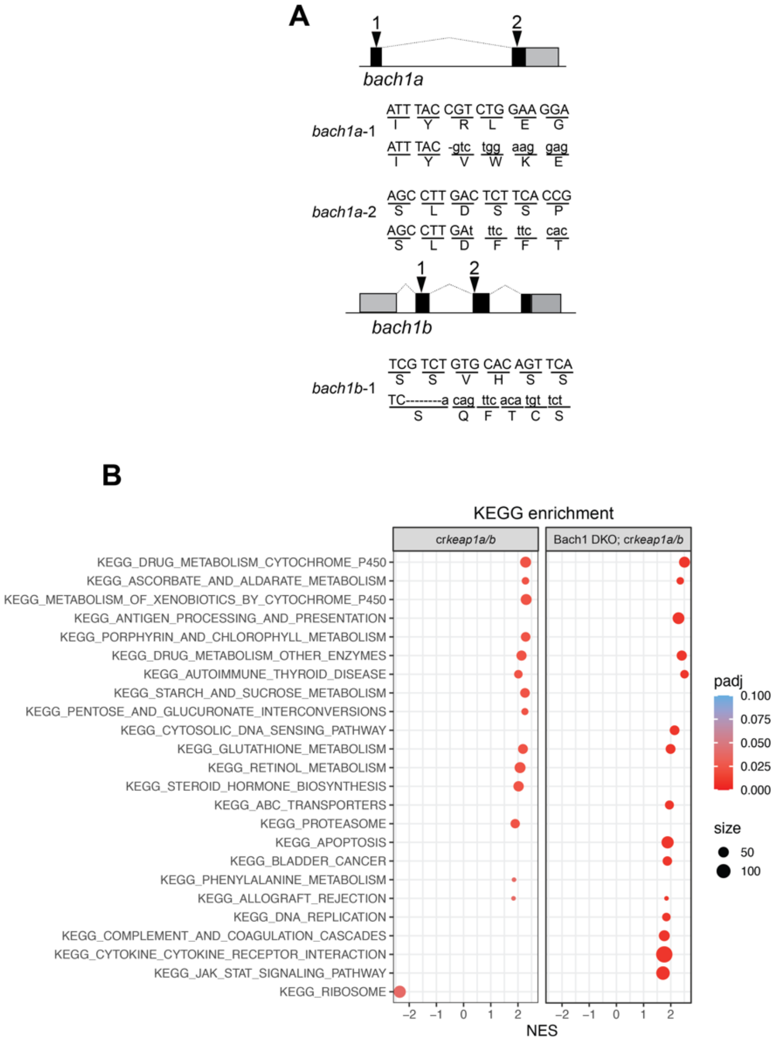

Fig. S4. Bach1-mediated repression of Nrf2 modulates lysosomal biogenesis.

(A) Schematic of mutations in the Bach1 DKO zebrafish line. (B) Bubble plot of KEGG pathways enriched in GSEA of cr*keap1a/b* versus WT and Bach1 DKO; cr*keap1a/b* versus Bach1 DKO at 7 dpf.

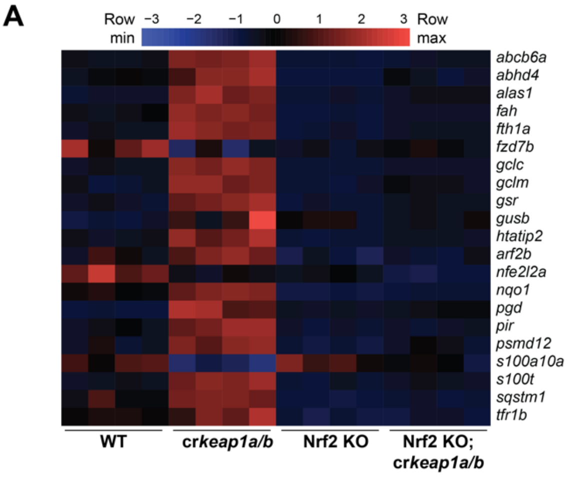

Fig. S5. Keap1-dependent regulation of lysosomal biogenesis requires Nrf2.

(A) Heatmap of Nrf2 target gene expression in WT, cr*keap1a/b*, Nrf2 KO, and Nrf2 KO; cr*keap1a/b* zebrafish at 7 dpf as determined by RNA-Seq analysis.

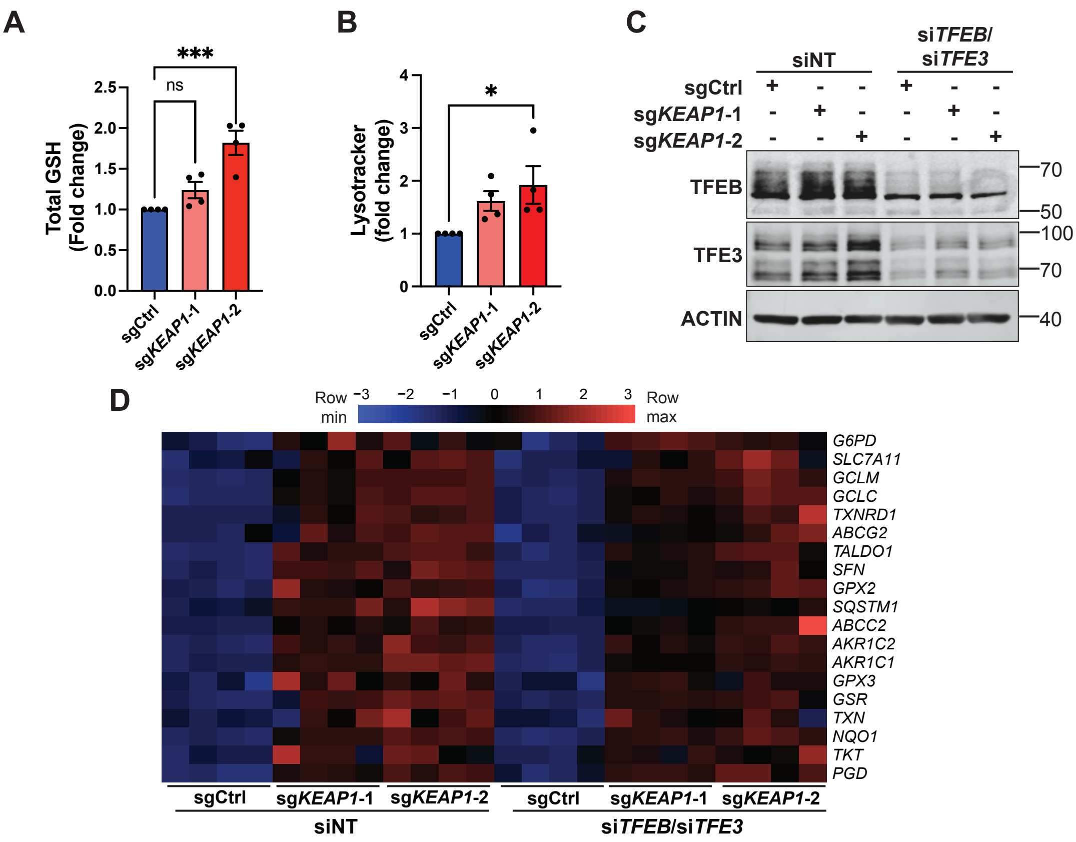

E

Cancer Type	PRDX1 WITH ATP6V0B		PRDX1 WITH GLA		TXN WITH ATP6V0B		TXN WITH GLA	
	Spearman	*p*-value	Spearman	*p*-value	Spearman	*p*-value	Spearman	*p*-value
ACC	0.28	1.45E-02	0.09	4.25E-01	0.20	8.41E-02	0.27	1.93E-02
BLCA	0.47	1.00E-23	0.35	3.00E-13	0.24	1.50E-06	0.33	1.14E-11
BRCA	0.56	7.44E-85	0.12	8.91E-05	0.42	5.68E-43	0.15	2.35E-06
CESC	0.56	9.62E-24	0.32	4.73E-08	0.25	2.31E-05	0.26	9.58E-06
CHOL	0.59	1.45E-04	0.39	2.02E-02	0.39	1.86E-02	0.48	3.07E-03
COAD	0.47	6.66E-30	0.34	2.37E-15	0.20	3.04E-06	0.29	1.25E-11
DLBC	0.72	4.65E-07	0.65	1.12E-05	0.54	5.06E-04	0.57	2.60E-04
ESCA	0.26	3.72E-04	0.35	1.02E-06	0.02	7.58E-01	0.30	3.44E-05
GBM	0.53	5.17E-12	0.35	1.39E-05	0.47	2.04E-09	0.42	1.63E-07
HNSC	0.51	3.61E-33	0.48	7.35E-29	0.32	3.65E-13	0.32	6.00E-13
KICH	0.16	2.02E-01	0.38	1.55E-03	0.41	6.77E-04	0.12	3.26E-01
KIRC	0.59	6.07E-35	0.59	1.30E-34	0.40	3.09E-15	0.28	1.56E-07
KIRP	0.49	1.05E-17	0.57	3.84E-25	0.51	2.54E-19	0.52	1.67E-20
LAML	-0.04	6.07E-01	-0.17	2.85E-02	0.13	9.05E-02	0.28	2.40E-04
LGG	0.49	7.41E-32	0.29	1.58E-11	0.60	3.07E-51	0.39	8.37E-20
LIHC	0.64	1.97E-41	0.49	3.19E-22	0.39	6.34E-14	0.37	6.58E-13
LUAD	0.41	1.34E-21	0.47	2.83E-29	0.25	1.29E-08	0.40	2.87E-20
LUSC	0.22	1.02E-06	0.61	1.99E-48	-0.07	1.46E-01	0.61	3.63E-49
MESO	0.67	4.13E-12	0.46	1.57E-05	0.38	4.11E-04	0.46	1.24E-05
OV	0.61	9.13E-22	0.39	1.40E-08	0.43	2.41E-10	0.37	5.71E-08
PAAD	0.39	1.71E-07	0.25	1.14E-03	0.19	1.46E-02	0.12	1.20E-01
PCPG	0.74	2.85E-29	0.11	1.49E-01	0.21	7.90E-03	0.27	4.46E-04
PRAD	0.57	2.30E-43	0.44	5.84E-24	0.41	2.01E-21	0.34	1.01E-14
SARC	0.75	1.09E-45	0.56	3.38E-22	0.66	3.84E-33	0.54	2.51E-20
SKCM	0.41	3.51E-16	0.30	4.39E-09	0.34	4.14E-11	0.26	4.91E-07
STAD	0.60	2.14E-40	0.26	1.09E-07	0.37	9.54E-15	0.29	3.49E-09
TGCT	0.80	9.02E-34	0.48	1.50E-09	0.59	6.95E-15	0.41	3.11E-07
THCA	0.37	1.24E-16	0.31	7.15E-12	0.37	1.24E-16	0.31	7.15E-12
THYM	0.32	3.44E-04	-0.07	4.41E-01	0.67	1.54E-16	0.78	2.24E-25
UCEC	0.29	3.70E-11	0.38	3.52E-19	0.20	5.45E-06	0.32	1.31E-13
UCS	0.69	3.27E-09	0.29	3.16E-02	0.54	1.69E-05	0.48	2.03E-04
UVM	0.36	2.81E-20	0.35	9.34E-19	0.18	8.92E-07	0.20	6.22E-07

Fig. S6. KEAP1-dependent regulation of lysosomal biogenesis is cell-autonomous and evolutionarily conserved.

(A) Quantification of glutathione (GSH) content in sg*KEAP1*-1, and sg*KEAP1*-2 HepG2 cells relative to sgCtrl cells. Data are shown as mean ± SEM, n = 4. (B) Quantification of Lysotracker fluorescence intensity in sg*KEAP1*-1, and sg*KEAP1*-2 HepG2 cells relative to sgCtrl cells. Data are shown as mean ± SEM, n=4. (C) Representative

immunoblot analysis of sgCtrl, sg*KEAP1*-1, and sg*KEAP1*-2 HepG2 cells transfected with non-targeting siRNAs (siNT) or siRNAs targeting *TFEB* and *TFE3* (si*TFEB*/si*TFE3*). (D) Heatmap of NRF2 target genes in sgCtrl, sg*KEAP1*-1, and sg*KEAP1*-2 HepG2 cells transfected with siNT or si*TFEB*/si*TFE3* as determined by RNA-Seq analysis. (E) Spearman's rank-order correlation of expression of NRF2 target genes *PRDX1* and *TXN* with the lysosomal genes *ATP6V0B* and *GLA* in TCGA datasets.

Materials and Methods

Zebrafish CRISPR/Cas9 gene editing

Global editing via CRISPR/Cas9 was performed as previously described (1, 2). Two independent Alt-R CRISPR-Cas9 crRNAs (Integrated DNA Technologies) targeting *keap1a*/*keap1b*/*bach1a*/*bach1b*, and combinations thereof, were employed. Guides were complexed with recombinant Alt-R® S.p. Cas9 Nuclease V3 (Integrated DNA Technologies) *in vitro* prior to microinjection. Cas9-guide complex reactions contained 24 µM guide RNA, 24 µM Alt-R® CRISPR-Cas9 tracrRNA (Integrated DNA Technologies) and 5 µg Cas9 in a total volume of 5 µL. Cas9-guide complexes were injected into the yolk of one-cell stage zebrafish embryos.

Table 1. Target sites for gene knockout with CRISPR/Cas9 in zebrafish

Gene	Site	Target site
keap1a	1	GTAACCGTGACCCCTCATGG
	2	CTTCAAGGAGTGCCACGCGT
keap1b	1	ACCAACGGCCTAAAGGAGTG
	2	CTGGACCCCAGTAACGCCAT
bach1a	1	GTTAGACTGCATTTACCGTC
	2	CGGACTGCGGTGAAGAGTCA
bach1b	1	GTGAACTGTGCACAGACGAC
	2	CTTCACCAAAGAGAACGTGC
flcn	1	TTTGTGAGCTCCATGGCCCA
	2	ATCAAAGACAACCTGGCACG

Assessment of gene editing efficiency

Genomic DNA was extracted from injected zebrafish larvae using DirectPCR lysis reagent (Viagen) with proteinase K (Thermo Fisher Scientific). PCR was performed using KOD Hot Start Polymerase (Sigma-Aldrich). Sanger sequencing was performed on gel purified amplicons by the Australian Genome Research Facility (AGRF). Indels in the sequenced data were analysed using the Inference of CRISPR Edits (ICE) web-based platform (Synthego).

Table 2. Primers utilized for ICE sequencing

ICE sequencing primer name	Primer sequence
bach1a.1-F	CCCAACTCTTGGCTGAACAT
bach1a.1-R	AACCGAAGGCATGATAATCG
bach1a.2-F	GTTTGCGCGGCTATTTACTC
bach1a.2-R	AAGGCTTAGTCGCATTTCCA
bach1b.1-F	AGAGAGTCGGAGCAAGCAAA
bach1b.1-R	AACTTGGTCATGGAGGGTTG
bach1b.2-F	GAGGAAAAGCCCAGTGTGAA
bach1b.2-R	CCCACAAGCGATCTGAAACT
keap1a.1-F	CACGTGGATGGGTATAGACATTGT
keap1a.1-R	CAGTAAACCACAAAGCTGTCACC

keap1a.2-F	GGTGTGTTTCTGCCTGTTTCTTTT
keap1a.2-R	AGTGGCATGAGGCTGAACAAC
keap1b.1-F	GTACGCAAACTCAATGAGCCGG
keap1b.1-R	GCACCGCGTCTTCAGCTACA
keap1b.2-F	GACTGCACACTGCGCTGTACA
keap1b.2-R	CCCACTTCAGAAACGGTGTGTG
flcn.1-F	AAATGCTGCACTTCCCATCG
flcn.1-R	CCATCCACACTCACAAAGCC
flcn.2-F	CCGCAAAATAAACCATGAAACTTAT
flcn.2-R	TGTCCCACAGATTGGGATGC

Multiphoton microscopy

Zebrafish larvae were anesthetized in E3 medium containing 0.2 mg/mL Tricaine and embedded in agarose larval arrays. Live imaging was performed on an Olympus FVMPE-RS multiphoton microscope using a 25x objective equipped with Olympus FV30-SW software. Images were analyzed and parameters were quantified using the 'Spot' and 'Surface' functions on Imaris v9.3 (Oxford Instruments).

Histology

Zebrafish larvae were fixed in 4% paraformaldehyde and embedded in agarose larval arrays. After paraffin-embedding and serial sectioning, slides were stained with hematoxylin and eosin (H&E) using a previously published protocol (3). Sections were imaged with an Olympus BX53 microscope.

Transmission Electron Microscopy

Zebrafish larvae were anesthesized in E3 medium containing 0.2 mg/mL tricaine before incubation in fixative solution containing 2.5% glutaraldehyde and 2% paraformaldehyde in 0.1 M cacodylate buffer for 2h at RT. Larvae were washed three times in 0.1 M cacodylate buffer before post-fixation, dehydration, and embedding. Samples were embedded in Spurr's resin (Sigma-Aldrich) for sectioning. Ultrathin transverse sections of the liver were prepared with a diamond knife (Diatome) using a Leica Ultracut E before mounting on copper grids. The sections were stained with aqueous uranyl acetate and aqueous lead citrate as contrasting agents. Sections were imaged with a JEOL 1400 transmission electron microscope.

RNA extraction and RNA-Seq analysis

For RNA-Seq analysis of zebrafish larvae, larvae were pooled (n=10), anesthesized in E3 medium containing 0.2 mg/mL Tricaine, and homogenized in TRIzol™ (Thermo Fisher Scientific). For RNA-Seq analysis of zebrafish larval livers, livers were isolated by dissection under an SZX12 microscope equipped with DFPLapo1XPF (X-Cite 120Q) (Olympus). Dissected livers were pooled (n=15) and transferred to TRIzol™. RNA was extracted using the Direct-zol™ RNA MiniPrep Kit (Zymo Research) according to manufacturer's instructions. For HepG2 cells, RNA was extracted using the NucleoSpin RNA kit (Macherey-Nagel) as per manufacturer's instructions. In all cases, RNA quality was confirmed using an Agilent 4200 TapeStation System. Libraries were prepared using the QuantSeq 3' mRNA-Seq kit (Lexogen) and sequenced with an Illumina NextSeq 500, with single-end 75bp reads to a depth of 5M reads per sample. FASTQ files were uploaded to the Galaxy web platform for quality control (FastQC), trimming (Cutadapt), alignment (RNA STAR), and counting (featureCounts) (4). For zebrafish samples, the reads were aligned to the zebrafish reference genome (Ensembl danRer11, GRCz11) and annotated with the GTF from Lawson et. al. (5). Reads from HepG2 cells were aligned to the human genome assembly (Ensembl hg19, GRCh37). Analysis of differentially expressed genes was performed with Limma-Voom (v3.40.6). Gene set enrichment analysis (GSEA) was performed and plotted in R

using the ‘fgsea’ package (6). Heatmaps and volcano plots were generated in R using the ‘pheatmap’ and ‘ggplot’ packages respectively.

Immunofluorescent staining of larval sections

Immunofluorescence on larval sections was performed as previously described (7). Stained sections were imaged with an Olympus FV3000 confocal microscope using a 60x objective equipped with Olympus FV30-SW software.

ß-galactosidase (ß-gal) staining

ß-gal staining was performed as previously described for zebrafish larvae (8) and cultured cells (9). For zebrafish, larvae were fixed in 4% PFA for 1 h at room temperature. After PBS washing, larvae were incubated overnight at 37°C in ß-gal staining solution containing 1 mg/mL 5-bromo-4-chloro-3-indolyl ß-D-galactoside/X-Gal (Thermo Fisher Scientific), 40 mM citric acid/sodium phosphate at pH 6.0, 5 mM potassium ferricyanide, 5 mM potassium ferrocyanide, 150 mM sodium chloride, and 2 mM magnesium chloride. Zebrafish larvae, and dissected livers, were imaged with a NSZ-606 Zoom Stereomicroscope and images were captured using TCapture(IS Capture) (Tucsen Photonics Co.). For liver sections of ß-gal stained larvae, larvae were stained with ß-gal before paraffin-embedding and sectioning. The sections were de-paraffinized and mounted with DPX before imaging. For HepG2 cells, cells were fixed with 4% PFA at room temperature for 10 min. Cells were subsequently stained with ß-gal staining solution at 37°C for 24 h. Brightfield images were taken with a Zeiss Axiovert microscope.

Generation of KEAP1 knockout HepG2 cell lines

Guide RNA target sequences were determined using the GPP sgRNA Designer (Broad Institute). Guide RNAs for *KEAP1*, or a control targeting *AAVS1*, were cloned into the LentiCRISPRv2 vector (a gift from Feng Zhang, Addgene plasmid #52961) as previously described (10, 11). Lentiviral particles were generated by co-transfecting 293T cells with pLentiCRISPv2 vectors (3rd generation lentiviral vector), pCMV-VSV-G (a gift from Bob Weinberg, Addgene plasmid #8454) and psPAX2 (a gift from Didier Trono, Addgene plasmid #12260). HepG2 cells were transduced with lentiviral particles and selected with puromycin (3 µg/mL).

Table 3. Target sites for gene knockout with CRISPR/Cas9 in HepG2 cells

Gene	Guide	Target site
KEAP1	1	ACAACCCCATGACCAATCAG
	2	TTGGCATCATGAACGAGCTG
AAVS1	168	CCAGCCGTAGAGGTGACCC

Immunoblotting

For the collection of whole cell lysates, cells were washed with PBS and lysed in SDS lysis buffer (1% SDS, 50 mM Tris-HCl, 10 mM EDTA) containing a protease inhibitor cocktail (Sigma-Aldrich) and Pierce™ Universal Nuclease (ThermoFisher Scientific). For the collection of nuclear fraction lysates, cells were trypsinized and resuspended in Buffer A (pH 7.0, 10 mM HEPES, 5 mM $MgCl_2$, 25 mM KCl) at 1×10^4 cells/µL. Cell suspension was passed through a 26-gauge needle twenty times and incubated on ice for 15 min. NP-40 was added to each sample to attain 0.2% final concentration. Cells were vortexed for 10 s and centrifuged at 14,000g for 1 min at 4 °C. The supernatant was removed, while the nuclear pellet was lysed in 1 × SDS buffer (5% β-mercaptoethanol) at 1.4×10^4 cells/µL and boiled at 95 °C for 12 min. Lysates were resolved by SDS-PAGE and transferred to nitrocellulose membrane followed by immunoblotting. Membranes were incubated with IRDye secondary antibodies (LI-COR) and imaged using the Odyssey DLx imaging system (LI-COR).

Table 4. Primary antibodies utilized in this study

Antibody	Source	Cat. number
NRF2 (D1Z9C) XP® Rabbit mAb	Cell Signaling Technology	12721S
KEAP1 (D6B12) Rabbit mAb	Cell Signaling Technology	8047S
TFEB (D2O7D) Rabbit mAb	Cell Signaling Technology	37785
TFE3 Polyclonal antibody	ProteinTech	14480-1-AP
Histone H3	Abcam	ab1791
β-Actin (8H10D10) Mouse mAb	Cell Signaling Technology	3700
Anti-PCNA (IN), Z-FISH®	AnaSpec	55421

Gene knockdown with siRNA

HepG2 cells were reverse transfected with Dharmafect (Dharmacon, DHA-T-2001-03) according to manufacturer's instructions with pooled siRNAs targeting *TFEB* (Human ON-TARGETplus Human TFEB (7942) siRNA SMARTpool), *TFE3* (Human ON-TARGETplus Human TFE3 (7030) siRNA SMARTpool) or On-TARGETplus Nontargeting Control Pool (Dharmacon). Transfected cells were harvested 72 h after seeding.

Flow Cytometry

HepG2 cells were incubated in fresh media containing Lysotracker™ Red DND-99 (1:10000 dilution, ThermoFisher Scientific) for 1 h or Magic Red (Abcam) for 30 min. Cells were trypsinized and resuspended in FACS buffer (2% FBS in PBS). Cell suspensions were filtered through a 35 μm mesh before analysis on a BD LSRFortessa™ X-20 Cell Analyzer (BD Biosciences). Samples were analysed using FlowJo software (BD Biosciences).

Metabolite extraction and metabolomics analysis

Ten larvae were pooled, anesthetized, and snap frozen in 100 μL of E3 medium containing 0.2 mg/mL Tricaine before 600 μL of ice-cold 80:20 methanol:water extraction solvent was added. Samples were homogenized using a micro pestle homogenizer (ThermoFisher Scientific). Debris was pelleted by centrifugation and supernatant fractions were analysed by hydrophilic liquid chromatography (HILIC) and mass spectrometry (Agilent 6545 LC/Q-TOF). QC and peak alignment were performed using Agilent MassHunter software. A volcano plot was generated using the 'ggplot' package in R.

Gene expression correlation analysis

The correlations between NRF2 target genes (*PRDX1* and *TXN*) and TFEB/TFE3 target genes (*ATP6V0B* and *GLA*) were analysed using cBioPortal (http://www.cbioportal.org/), based on the RNA-Seq datasets from the TCGA database.

References for SI Appendix

1. S. E. DiNapoli, *et al.*, Synthetic CRISPR/Cas9 reagents facilitate genome editing and homology directed repair. *Nucleic Acids Res.* **48**, e38 (2020).
2. S. Vaidyanathan, *et al.*, YAP regulates an SGK1/mTORC1/SREBP-dependent lipogenic program to support proliferation and tissue growth. *Dev. Cell* **57**, 719-731.e8 (2022).

3. A. G. Cox, *et al.*, Yap reprograms glutamine metabolism to increase nucleotide biosynthesis and enable liver growth. *Nat. Cell Biol.* **18**, 886–896 (2016).
4. E. Afgan, *et al.*, The Galaxy platform for accessible, reproducible and collaborative biomedical analyses: 2016 update. *Nucleic Acids Res.* **44**, W3–W10 (2016).
5. N. D. Lawson, *et al.*, An improved zebrafish transcriptome annotation for sensitive and comprehensive detection of cell type-specific genes. *Elife* **9**, e55792 (2020).
6. G. Korotkevich, *et al.*, Fast gene set enrichment analysis. *bioRxiv* (2016) https:/doi.org/10.1101/060012.
7. M. Sande-Melón, *et al.*, Adult sox10+ cardiomyocytes contribute to myocardial regeneration in the zebrafish. *Cell Rep.* **29**, 1041-1054.e5 (2019).
8. S. Da Silva-Álvarez, *et al.*, Developmentally-programmed cellular senescence is conserved and widespread in zebrafish. *Aging* **12**, 17895–17901 (2020).
9. K. T. Chan, *et al.*, Combining high-content imaging and phenotypic classification analysis of senescence-associated beta-galactosidase staining to identify regulators of oncogene-induced senescence. *Assay Drug Dev. Technol.* **14**, 416–428 (2016).
10. N. E. Sanjana, O. Shalem, F. Zhang, Improved vectors and genome-wide libraries for CRISPR screening. *Nat. Methods* **11**, 783–784 (2014).
11. O. Shalem, *et al.*, Genome-scale CRISPR-Cas9 knockout screening in human cells. *Science* **343**, 84–87 (2014).
